# Self-Assembly of Cellulose Nanocrystals and Organic Colored Pigments as Reinforcement Matrix of Lipstick for Enhancing SPF

**DOI:** 10.1155/2022/2422618

**Published:** 2022-02-09

**Authors:** Lidan Xiong, Hailun He, Jie Tang, Qi Yang, Li Li

**Affiliations:** ^1^Cosmetics Safety and Efficacy Evaluation Centre, Sichuan University West China Hospital, 610041, China; ^2^Sichuan Engineering Technology Research Centre of Cosmetic, 610041, China; ^3^Department of Dermatology, Sichuan University West China Hospital, 610041, China; ^4^Department of Polymer Science and Engineering, Sichuan University, 610041, China

## Abstract

The vermilion of the human lip, covered by a skinny epithelium with little melanin, is quite susceptible to damage from ultraviolet (UV) radiation exposure. However, commercial sunscreen filters and indelible dyes used in lipsticks can cause health hazards after percutaneous absorption or accidentally oral administration. Inspired by plant pigmentation as natural filters to protect themselves against overexposure to UV, safer bio-based sunscreens of cellulose enveloped with anthocyanin (AN) were developed using bionic design. Cellulose nanocrystals (CNC), derived from acid hydrolysis of cellulose, reinforced enhancement of UV absorption and shielding properties of AN. This innovation addresses the issue that naturally sourced UV filter application to sunscreen does not achieve a desired sun protection factor (SPF) value because of the low specific extinction value (E1,1). We also stated that the diverse formula of anthocyanin sunscreen lipsticks with CNC exhibited 10 times more SPF value than AN alone. Furthermore, they possess competitive benefits such as pleasing texture, superior adhesion, impermeable, nonphototoxicity, ease of application, and removal. This work provides a promising proof-of-concept for studying the features of natural sunscreens in the design of simple, safe, efficient, and green sunscreens.

## 1. Introduction

Chronic exposure to ultraviolet (UV) radiation is known to damage the structure and function of the skin. It has long been recognized that using artificially synthesized sunscreens to protect the skin as long ago as the 19th century [1]. As recently as 1985, the significance of UV-induced lip cancer was recognized [2, 3]. The lips are covered by a very thin orthokeratotic horny layer, containing little melanin. Thus, they are highly sensitive to chemical and physical insults, especially UV-exposed lip skin. In particular, UV radiation damages human lips, including photoaging, actinic cheilitis, and malignant tumor [4, 5]. In comparison, oral exposure to UV radiation is associated with a higher risk of cancer than skin exposure [6].

**Figure 1:**
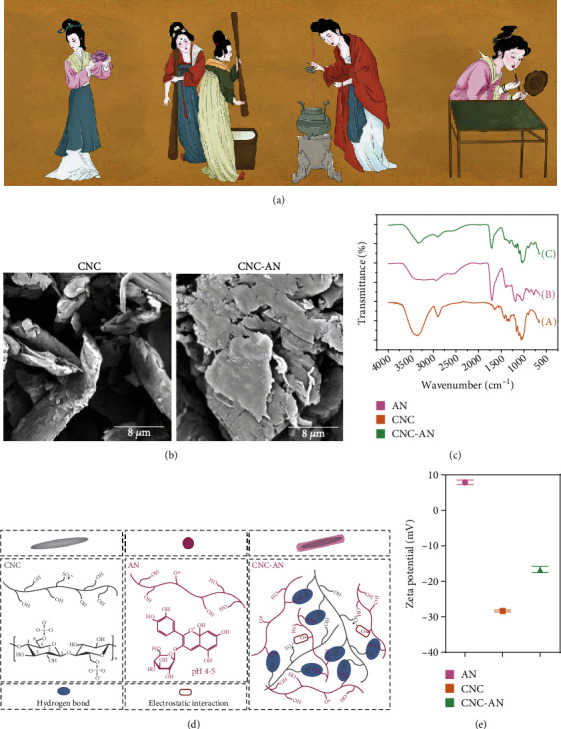
(a) The lipstick made by traditional ancient methods. (b) Scanning electron microscopic (SEM) images of CNC and CNC-AN. (c) The Fourier transform infrared spectrometer (FTIR) was applied to evaluate the CNC, AN, and CNC-AN. (d) The synthetic scheme toward CNC and AN via electrostatic adsorption and hydrogen bond. (e) Zeta potential of CNC, AN, and CNC-AN.

Dating back to ancient Egypt and the Tang Dynasty of China, traditional rouge, roughly equivalent to modern lipstick, was formulated from nontransparent minerals and plants such as clove and cinnabar (Figure 1(a)). Currently, the sunscreen filters in lipstick consist primarily of artificial chemicals, such as oxybenzone, avobenzone, 4-methylbenzylidene camphor, octocrylene, and inorganic filters, such as titanium dioxide and zinc oxide [7]. However, there are some controversies and side effects associated with these agents, which can penetrate through the epidermis or even be absorbed into the bloodstream leading adverse health effects on humans [8–12]. Specifically, the risk of synthetic UV actives included in lipstick is increased when they are accidentally taken orally. In general, natural sunscreens are likely to exhibit fewer detrimental side effects.

During the path of evolution, plants have evolved efficacious safeguard mechanisms against overexposure to solar radiation. Flavonoids, especially anthocyanin (AN), are the main anti-UVB polysaccharides that have demonstrated chemopreventive effect via *π*-stacking interactions [13]. Moreover, anthocyanin shows color variations under different pH values [14], which can create color reviver balm because of different pH values in human lips [15]. Previous studies have documented that many polyphenols with aromatic moieties possess anti-UV radiation activity, but the inherent variation in bioactivity, concentration, and specific extinction value (E1,1) poses some shortcomings on the application in sunscreen products [15]. Currently, none of the reported botany sunscreen exhibit sun protection factor (SPF) values higher than 30 [16]. To overcome this challenge, it is therefore urgent to develop a safe and effective sunscreen lipstick so as to avoid hazardous chemical filters and dyestuff constituents.

In this work, our effort was focused on design and preparation of CNC-AN lipstick sunscreens that were safe and simple for human lip photoprotection. Inspired by natural fibers of textile products, cellulose from hemp stalk enveloped with AN UV absorbers in the vacuoles was developed using bionic design [17]. Binding of anthocyanins to acid hydrolysis of cellulose, the lipstick exhibited pleasing texture due to the reddish color. Remarkably, mimicking native plant cell wall binding with AN could lead to a significant increase of UV-adsorption, UV-shielding, and SPF value, probably because UV-filters binding with high surface area cellulose changed the physical properties of organic particulate UV absorbers such as size or light path [18]. Further, the sunblock lipstick based on AN-CNC contained none commercial UV-filters. The novel biopolymer sunscreen lipsticks, avoiding ingesting synthetic sunscreen and dyestuff constituents, were fulfilling food-grade, impermeable, and nonphototoxicity.

## 2. Material and Methods

### 2.1. Materials

Red cabbage (*Brassica oleracea L. var capitata f. rubra*) was purchased from a local supermarket, while Pectinase and Cyanidol-3-Glucoside were obtained from Aladdin (Shanghai, China). Hemp stalk was provided as a present. In addition, cationic hydroxyethyl cellulose (cHEC), CCK-8 (Cell Counting Kit-8), fluorescein isothiocyanate (FITC), and 2′,7′-dichlorohydrofluorescein diacetate (DCFH-DA) were acquired from Usolf (Qingdao, China), Dojindo (Dojindo Laboratories, Japan), and Sigma Chemical Co. (Sigma, USA), respectively. Anti-*γ*H2AX was obtained from Abcam (Abcam, UK). DMEM, penicillin, and streptomycin solution were purchased from HyClone (GE Health Care Life Science, USA), while B27 was obtained from Gibco (Thermo Fisher, USA). BALB/c mice and nude mice were purchased from Dashuo Biological Technology Co., Ltd. (Chendu, China).

### 2.2. Methods

#### 2.2.1. Preparation and Characterization of CNC-AN

*(1) Preparation of CNC-AN Homogenate and Freeze-Dried Powders*. CNC extracted using acid hydrolysis of hemp stem was added into anthocyanin liquid at 0.15 g/mL and then stirred at 60°C for 30 min. Then, the cellulose was centrifuged at 2000 rpm for 15 min [19]. The precipitation was grafting CNC-AN homogenate and eventually freeze-dried.

*(2) Morphological and Fourier Transform Infrared (FTIR) Examination*. CNC and CNC-AN that were taken were subjected to morphological study using scanning electron microscopy (SEM) Phenom Pro (phenom world, Netherlands) at an acceleration voltage of 10 kV. Then, we compared CNC-AN with CNC and AN using FTIR (FT-IR, Nicolet 6700, Thermo Fisher Scientific, USA).

*(3) UV Absorption and Shielding Determination*. All band ultraviolet scanning for measuring UV absorption was determined using a spectrophotometer (Thermo Fisher, USA). We detected UV shielding rate using UV-LED light (Sigma, USA) and UV irradiance meter (National Institute of Metrology, China).

*(4) Zeta Potentials and Bioadhesion Determination*. Zeta potentials of CNC, AN, and CNC-AN were detected using Zeta View (Particle Metrix, Germany). To observe the bioadhesion, CNC, AN, and CNC-AN were smeared on the inner forearm and took pictures for 15 sec continuously.

#### 2.2.2. Fabrication and Characterization of CNC-AN Lip Gloss and Lipstick

For this procedure, we briefly added 0.5 g CNC-AN freeze-dried powder into 6.6 g soft oil. After evenly dispersed, we added 2.1 g hard oil and stirred it at 120°C. When the hard oil was completely melted, we then poured it into the mold to formulate lipstick [20]. Additionally, CNC-AN, AN, and cHEC were mixed at ratios of 10 : 10 : 1, stirred evenly at 60°C, and then cooled down to room temperature to obtain lip gloss. The stability of lipstick and lip gloss was studied at 40°C and -15°C. The analyses were conducted placing samples of lipstick and lip gloss (final pH 6.5–6.8) in an electrothermal incubator or refrigerator for 24 h. In order to explore the stability of formulations for 3 months, lipstick and lip gloss were kept in stability cabinet (Shang Hai Jinhong, China) 50 ± 0.1°C and 60% relative humidity and in refrigerator −5 ± 0.1°C. Finally, we captured SEM images of CNC-AN, lip gloss, and lipstick using Phenom Pro (phenom world, Netherlands) at an acceleration voltage of 10 kV.

#### 2.2.3. In Vitro Evaluation

*(1) SPF Determination*. The SPF value of the lipstick and lip gloss was assessed by SPF 290S (Optometric, USA) [21]. The sample was sprinkled on the 3 M film with a needle syringe (2 mg/cm^2^ sample layer). Then, it was placed at room temperature for 30 minutes in the dark for SPF determination.

*(2) Cell Culture*. Here, the human keratinocyte cell line (HaCaT) was acquired from China Center for Type Culture Collection (Kunming, China). HaCaT was cultured in RPMI DMEM media (Dulbecco's Minimum Essential Medium), supplemented with 100 U/mL penicillin, 100 *μ*g/mL streptomycin, and 10% FBS (Gibco). Then, the cells were incubated at 37°C in 5% CO_2_ [22].

*(3) Cell Viability Assay*. HaCaT (3.0 × 10^4^ cells/well) were seeded into 96-well plates. Cultures were preprocessing with varying concentrations of anthocyanin for 24 h. Subsequently, we added CCK-8 solution to each well for 3 h. Absorbance was measured at 450 nm using a microplate spectrophotometer (Bio-Rad Inc., USA) [22].

*(4) Protection of UVB-Induced Oxidation Impact In Vitro*. The intracellular reactive oxygen species (ROS) level, which can be measured with the fluorescence intensity of DCFH-DA, was applied to evaluate antioxidant ability [22]. HaCaT was incubated in the 6-well plate and was assigned to control, UVB, sunscreen, lipstick, and lip gloss groups, respectively. The sunscreen, lipstick, and lip gloss were applied on a slide that can penetrate UVB, whereas HaCaT was exposed to 60 mJ/cm^2^ UVB leading to 60% cell viability. Then, cells were incubated with DCFH-DA for 60 min at 37°C. We finally recorded the fluorescence images using a fluorescent inverted microscope (Olympus IX71, Japan).

*(5) Protection of UVB-Induced DNA Damage In Vitro*. The DNA break in HaCaT cells can be detected using a comet assay [22]. First, place the first layer of the regular melting agar sugar (LMA) in the oven at 60°C. Then, 10 *μ*L cells and 75 *μ*L 0.7% low melting agar sugar (LMA) were the second layer of LMA at 4°C for 10 min to solidify. After lysis, electrophoresis, neutralization, and staining, the images were recorded using fluorescence microscopy. The percentage of tail DNA% was evaluated with CASP1.2.3 (Casplab, Poland). To evaluate the protection of UVB-induced nucleus damage by CNC-AN lipstick and lip gloss, *γ*H_2_AX cell immunofluorescence was applied [22]. HaCaT cells in 24-well plates were classified into control, UVB, sunscreen, lipstick, and lip gloss groups. Every group occupied 3 wells. After paraformaldehyde fixation, *γ*H_2_AX antibody, and secondary antibody incubation, the fluorescence images were recorded under a fluorescent inverted microscope (Olympus IX71, Japan).

*(6) Organotypic Cultures*. BALB/c mice were sacrificed using CO_2_ asphyxiation, and the lip was harvested and sterilized with 70% alcohol. We placed the lip clippings into cold PBS solution and cut the excess skin section off. Then, lip explants were incubated epidermal-side-up on a transwell in 6-well plates. The cell culture media consisted of DMEM (low glucose), supplemented with B-27, 25 units/mL penicillin, and 25 mg/mL streptomycin. Organotypic cultures were maintained in a 5% CO_2_ incubator at the temperature of 37°C for 24 h. Five wells were allocated to control, UVB, sunscreen, lipstick, and lip gloss groups, respectively. Then, lips were exposed to 5040 mJ/cm^2^ UVB. After irradiation, the lips were incubated in a 5% CO_2_ incubator at the temperature of 37°C for 72 h. The lips of mice were collected to stain hematoxylin and eosin (HE) and taken images by a microscope.

*(7) Transdermal Penetration in Pigskin*. FITC grafting freeze-dried powder was applied to prepare lipstick and lip gloss. 100 *μ*m thick lipstick and lip gloss were applied to the pigskin, while PBS with free FITC was used as positive control. After 6 h, the samples were frozen in a frozen section compound (FSC 22, Leica Microsystems, Buffalo Grove, USA) and vertically cut into 4 *μ*m thick slices using Cryostat microtome (Leica, Mainz, Germany) at -20°C [23]. Then, the sections were imaged under a microscope (Olympus, Tokyo, Japan), while the depth of fluorescence penetration was measured using ImageJ 7.0.

#### 2.2.4. Animal Experiments

*(1) Evaluation of Lipstick and Lip Gloss Skin Adhesion and Easy Wiping*. 0.1 g CNC-AN freeze-dried powder was dissolved in 3 mL DMSO, followed by the addition of 0.6 mL FITC/DMSO (0.01 g/mL) and 0.2 g Ditin butyl dilaurate (DBTDL). After a water bath at 100°C for 4 h, the reaction mixture was precipitated, dried, dialyzed, and frozen. Afterward, three nude mice were used to evaluate the adhesion of lipstick and lip gloss. FITC grafting freeze-dried powder was used to prepare lipstick and lip gloss (commercial lipstick as a positive control) [24]. Using IVIS Spectrum (Caliper Life Sciences) [25], we imaged and quantified lipstick, lip gloss, and commercial lipstick remaining on the skin at 0, 2, 4, and 6 h, before and after being washed with water or wiped a wet towel.

*(2) UV Radiation and Lipstick Treatment*. The animal experiments were approved by the ethics committee of West China Hospital following the National Institutes of Health guidelines for the care and use of laboratory animals. BALB/c mice were divided into three groups (*n* = 2 each), where 1 was for lipstick, while the other was for lip gloss. Then, the back of mice was divided into 4 sections (control, UVB, sunscreen with UVB, lipstick with UVB, and lip gloss with UVB, at 1 cm × 1 cm × 100 *μ*m film). Then, the mice were exposed to 5000 mJ/cm^2^ UVB. After irradiation, the mice of the 3 groups were observed for another 24, 48, and 72 h. In this subsection, the back skin and lips of mice were collected. After fixation, embedding, dewaxing, hydration, and rinse, the sections were immersed in hematoxylin and eosin staining solution. Morphology was photographed under a microscope.

#### 2.2.5. Human Evaluation

*(1) Preparation for Human Evaluation*. Twenty people aged 20-45 years from Chengdu were enrolled in this study. The human experiments were approved by the ethics committee of West China Hospital according to the National Institutes of Health guidelines for care.

*(2) CIE 3D Space System*. We used the CIE 3D space system to evaluate the adhesion of lipstick and lip gloss [26]. The lipstick, lip gloss, and commercial lipstick were applied to the skin. The values of *L*∗ and *a*∗ were detected at 0, 1, 2, 3, 4, 5, 6, 7, and 8 h.

*(3) Dermoscopy*. To discover whether the lipstick and lip gloss can be removed with water or a wet towel, the CBS-606 portable dermatoscope (Xiangmei Technology, Taiwan, China) was applied to observe the residue in the furrow [27]. Before using the presentation, water washing and wet towel inside the forearm were imaged.

*(4) VISIA-CR*. Furthermore, to discover whether the lipstick and lip gloss can be removed with water or a wet towel on the lip, the VISIA-CR (Canfield Scientific, New Jersey, USA) was applied to image the lip [28]. Then, before the presentation, water washing and wet towel were imaged. Finally, the values of *a*∗ and glossiness were measured using Image Plus Pro 7.0.

## 3. Results and Discussion

### 3.1. Characterization of CNC-AN

Inspired by plant pigmentation as natural filters to protect themselves against overexposure to UV, binding of AN to CNC was designed and prepared. Our previous study has applied CNC in foundation liquid [19]. Herein, we formulated traditional rouge from nontransparent minerals and plants such as clove and cinnabar (Figure 1(a)). We had been successfully attempted using either extracted AN from a variety of plants or commercialized AN (Supporting Information, Figure S1). The optimal ratio of AN and CNC for complex formation was determined (Supporting Information). The interaction between water-soluble AN and CNC occurred spontaneously and can bind to cellulose fibers up to 60% *w*/*w* (Figure S2). This finding suggests that the adsorption of AN on cellulose on plant cell walls can readily occur in real food systems whenever the plant tissues are processed or masticated [29]. The as-prepared CNC-AN appeared as biomacromolecule particles whose average diameter was greater than 5 *μ*m as depicted in Figure 1(b) and Figure S3. AN acted as a layer of jelly covering the surfaces of CNC, showing strong binding interaction.

In addition, AN was complexed with CNC via hydrophilic, hydrogen, and electrostatic bonding interactions to form complexity (Figure 1(e)). There was sensitivity to native charges in the association between negatively charged cellulose fibers and positively charged AN [30]. Given this, zeta potential was approached. Zeta potential for CNC, AN, and CNC-AN was determined to be +7.87 ± 0.65 mV, −28.36 ± 0.27 mV, and −16.68 ± 0.90 mV, respectively (Figure 1(c)). Additionally, the magnitude of negative zeta potential was increased by the addition of CNC, thus improving AN electrostatic stabilization [31–33]. After grafting AN to CNC, the bioadhesion of CNC-AN was clearly enhanced (Figure S4).

We further used FTIR to illustrate the type of molecular interactions between AN and CNC (Figure 1(d)). The characteristic peaks of CNC were around 3400, 2900, 1420, and 1020 cm^−1^, which can be attributed to the O-H stretching, C-H stretching, C-H bending, and C-O-C bending, respectively [34]. On the other hand, the characteristic peaks of AN were 1700, 1400, and 1190 cm^−1^, which can be attributed to the C=C stretching vibration of the aromatic ring, C-O specific angular deformation, and O-C stretching vibration of anhydroglucose ring, respectively [2]. Compared with AN, we found no obvious change that occurred in the band of CNC-AN at 1700 cm^−1^, but CNC-AN became more intensive between 1000 and 1700 cm^−1^. These changes perhaps may be attributed to the stretching vibration of the C-C bond of an aromatic ring in AN and the interactions between AN and CNC. Compared with CNC, the intensity of the peak at 3400 cm^−1^ was significantly weakened. These changes are likely to be associated with the formation of a hydrogen bond between the free hydroxyl group. These findings implied that AN formed strong physical and chemical interactions with CNC [35, 36].

### 3.2. Effect of CNC-AN on Specific Extinction Value, UV Absorption, and Shielding

**Figure 2:**
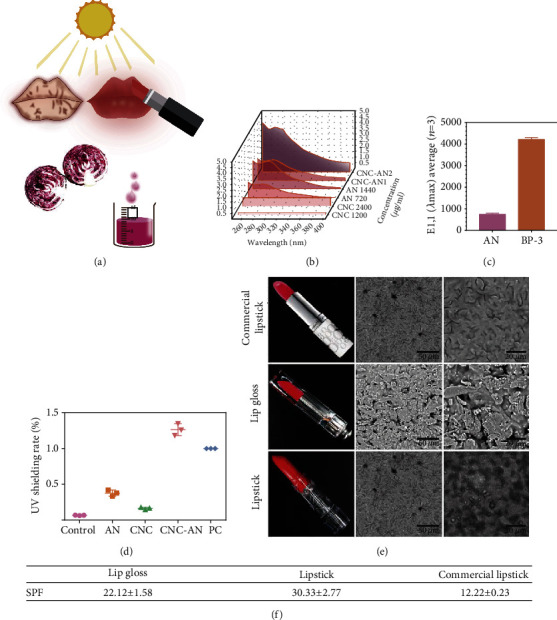
(a) Schematic design concept of sunscreen lipsticks. (b) The UV absorption of CNC, AN, and CNC-AN. (c) E1,1 (*λ*max) value of BP-3 and AN. (d) UV shielding rate of CNC, AN, CNC grafting AN, and BP-3 as a positive control. (e) Scanning electron microscopic (SEM) images of lip gloss, lipstick, and commercial lipstick. (f) Values of SPF of lip gloss, lipstick, and commercial lipstick.

To study the enhancing effects of UV absorbing properties of AN at different concentrations, we examined UV absorbing using a spectrophotometer. After binding CNC to commercialized AN, we recorded a twofold increase in the UV absorption compared with AN alone (Figure 2(b)). Likewise, the extraction of the AN from purple cabbage showed similar results (Figure S5). However, our findings indicated no increase in UV absorption of benzophenone-3 (BP-3, a commercial sunscreen) with CNC (Figure S5). Ultrapure water and one common sun-screening agent (BP-3) served as a negative and positive control, respectively, while BP-3 was included as a positive control. We noticed that resonance delocalization in BP-3 was aided by the presence of an electron-releasing group in either the ortho position, para position, or both, which results in *λ*max at 290 nm (Table S1 and Figure 2(b)). AN was composed of large *π*-conjugated domains with the formation of aromatic structures, which the maximum absorption wavelength (*λ*max) was characterized at 280 nm (Table S1 and Figure 2(b)).

Furthermore, one challenge here was that naturally sourced UV filter application to sunscreen did not achieve a desired sun protection factor (SPF) value because of the low specific extinction value (E1,1). As UV-filters, the specific extinction value (E1,1) is more important than UV-absorbance. We obtained the extinction E1,1 (*λ*max) using the following equation [16].

(1) $$\begin{array}{c} \text{E}1,1\;\left(\lambda \right)=\varepsilon \left(\lambda \right)\frac{10}{M}∙d, \end{array}$$

where *ε* refers to the molar decadic extinction coefficient, *M* is the molecular weight, and *d* denotes the optical pathlength. The extinction coefficient *ε* was calculated from the transmittance measurements according to the following equation.

(2) $$\begin{array}{c} E\left(\lambda \right)=\log\frac{1}{T\left(\lambda \right)}, \end{array}$$

where *T* refers to the transmittance.

(3) $$\begin{array}{c} \varepsilon \left(\lambda \right)=\frac{E\left(\lambda \right)}{c∙d}, \end{array}$$

where *E* denotes the extinction calculated via Eq, c in concentration in mol·L^−1^ and *d* is the optical pathlength in cm.

Our findings elucidated that the difference between BP-3 and AN in the E1,1 (*λ*max) value was about a 5-fold improvement (Table S1 and Figure 2(c)). Of note, E1,1 (*λ*max) presented the extinction E1,1 maximum wavelength, whereas *λ*max presented the wavelength of maximum absorbance. Elsewhere, both E1,1 (*λ*max) and *λ*max are two prime parameters that are identified to describe the performance of a UV-filter molecule [16].

In addition, using a UV light and a UV irradiance meter, the UV-shielding efficiencies of AN, CNC, CNC-AN, and BP-3 with 15 *μ*m thickness were irradiated. Our results uncovered that the standard sunscreen agent BP-3 was considered as 100% UV shielding, while that of CNC-AN homogenate was higher compared with the positive control, showing efficiency absorption > 126% UV light. Finally, after grafting CNC to AN, the UV shielding rate of the coupled AN tripled that of AN alone (Figure 2(d)).

### 3.3. Preparation and Characterization of Lipstick

Based on the improved UV shielding rate and ultraviolet absorption, we used CNC-AN homogenate to formulate sunscreen lipstick (Figure 2(e) and Table S2). Briefly, the homogenate was freeze-dried into a powder that was applied to formulate CNC-AN lipstick (commercial lipstick as positive control) (Figure 2(e)). Afterward, we herein employed two kinds of lip products, including cationic hydroxyethyl cellulose (cHEC) and diverse oils, to prepare the CNC-AN formula, and their resulting products were termed lipstick and lip gloss, respectively. In particular, lipstick was composed of CNC-AN (1 g), soft oil (6.6 g), and hard wax (2.1 g), as consistency in the traditional formula, while lip gloss comprised of CNC-AN complexed with cHEC hydrogels via hydrophilic, hydrogen bonding, and/or electrostatic interactions.

Then, the stability of formulations (lipstick/lip gloss) was studied. Both lipstick and lip gloss had red color. The pH of lipstick and lip gloss was range from 5 to 5.5 which were suitable for topical application. The result of stability studies showed that lipstick and lip gloss were stable during 3 months. The layering phenomenon was not present in lip gloss after returning to room temperature. The lipstick stayed hard after returning to room temperature.

The microscopic hierarchical structures of lip gloss, lipstick, and commercial lipstick were examined under a scanning electron microscope (SEM) (Figure 2(e)). The results highlighted that commercial lipstick exhibited a smooth structure, whereas lip gloss and lipstick containing CNC showed granular sensation (Figure 2(e)). The convex and flat profile belonged to the connection of CNC-AN to the substrate, yielding substantial interfacial adhesion. The TiO_2_ fillers were visible in commercial lipstick. Recent studies have established that long-term consumption of TiO_2_ is harmful to the body, such as disturbance of gut microbiota, intestinal inflammation, and immune response [37]. We also noted that lip gloss possesses lamellar microstructures with heterogeneity, while in lipstick, even fine particles dispersed.

### 3.4. Effect of Lipstick on *In Vitro* SPF

SPF value was detected using SPF 290S (2 mg/cm^2^ on 3 M film). The optometric model SPF-290 analyzer is a computer-controlled instrument that is designed to measure the SPF value of sunscreen preparations based on the US-FDA standards [38]. The collected data were plotted as an MPF (monochromatic protection factor) [38]. Our results of the SPF presented a very good correlation with the BASF sunscreen simulator presented, which exhibited a good correlation with real-world figures in *vivo* [38]. The SPF revalidated with 5% homosalate (HMS), 5% octocrylene (OCR), 4% 4-methylbenzylidene camphor (MBC), and 5% BP-3 hydroxyethyl cellulose hydrogel that are summarized in Table S3. Moreover, the values of SPF were 22.12 ± 1.58 for lip gloss, lipstick (30.33 ± 2.77), and commercial lipstick (12.22 ± 0.23), respectively (Figure 2(f)). These results suggest that lip gloss and lipstick possess high protection levels (usually corresponding to a measured SPF value > 15 for lipstick) as potential sunscreens [39]. Notably, the SPF value was improved 10-folder higher absorption after grafting CNC to AN compared with the AN alone. However, a similar amplification effect was not observed with chemical UV-filters. Overall, it is important to note that CNC can significantly improve the anti-UVB function of AN.

### 3.5. Bioadhesive Characteristics and Retention of Lipstick

**Figure 3:**
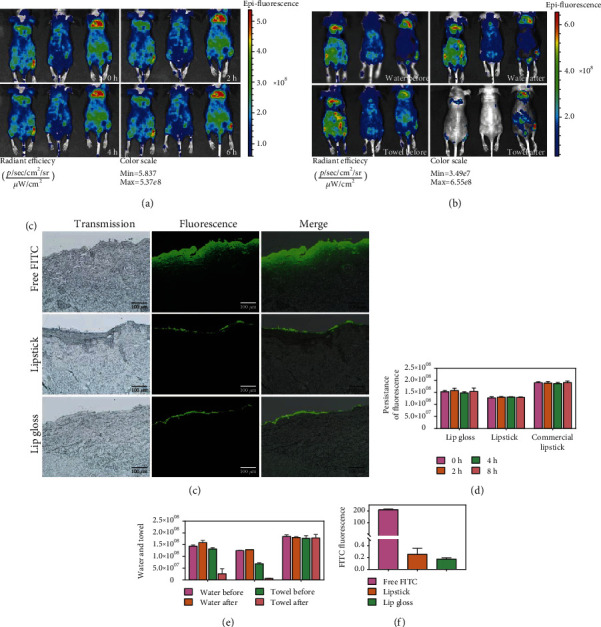
(a) IVIS images of the bioadhesion and retention of lip gloss, lipstick, and commercial lipstick at 0, 2, 4, and 6 h. (b) IVIS images of free FITC, lip gloss, and lipstick cleansing after washing with water or wiping with a wet towel. (c) Transdermal penetration of free FITC, lip gloss, and lipstick in pigskin. (d) The fluorescence intensity of bioadhesive and retention in lip gloss, lipstick, and commercial lipstick. (e) The fluorescence intensity of cleansing water and towel in lip gloss, lipstick, and commercial lipstick. (f) The penetration depth of free FITC, lip gloss, and lipstick.

We directly applied fluorescein isothiocyanate (FITC) to these samples by rubbing on the dorsal skin of nude mice, followed by imaging using an *in vivo* imaging system (IVIS). We observed no significant change in the fluorescent intensity among the three groups at 0, 2, 4, and 6 h (Figures 3(a) and 3(d)), revealing that the excellent bioadhesion and retention of lip gloss and lipstick were inconsistent with commercial lipstick. Likewise, the water resistance of the lip gloss and lipstick was evaluated using IVIS. The fluorescent intensity exhibited no change after water washing among the three groups, indicating good resistance against water. However, the lip gloss and lipstick were easy to wipe with a wet towel, while the commercial lipsticks were difficult to wipe (Figures 3(b) and 3(e)). Remarkably, easy wiping with a wet towel can decrease the use of facial cleanser, cleansing water, and cleansing oil, which is friendly to the skin particularly for those people with skin diseases [40, 41]. Furthermore, to identify the penetration behaviors, free FITC, lip gloss, and lipstick were applied to the pigskin, which was used as a surrogate for human skin [42]. After 6 h, the pigskin samples were frozen in a frozen section compound, and fluorescent depth was recorded (Figures 3(c) and 3(f)). We noted that free FITC penetrated the dermis, while lip gloss and lipstick did not penetrate the skin. These findings signify biosafety which is critical in the cosmetic industry [43]. Therefore, all characteristics including excellent bioadhesion, water resistance, easy wiping with a wet towel, and no penetration make CNC-AN gel and lipstick better products in the cosmetic industry.

### 3.6. Evaluation of Anti-UVB-Induced Oxidative and DNA Damage Activity *In Vitro*

**Figure 4:**
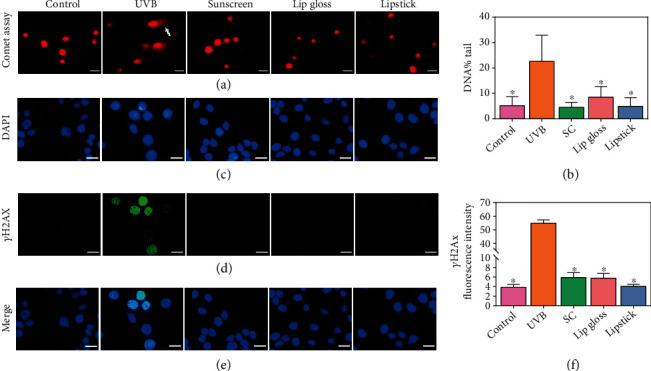
(a) The comet assay was applied to detect the DNA damage in HaCaT cells. (b) The statistical figure of DNA% tail. (c–e) *γ*H2AX cell immunofluorescence was applied to evaluate the nucleus damage. (f) The statistical figure of H2AX fluorescence level. ^∗^*P* < 0.05, the difference has statistical significance. Scale bars, 50 *μ*m.

To evaluate the safety of CNC-AN freeze-dried powder, we herein employed a cell viability assay which was detected using CCK-8 assay. Up to 10 mg/ml, the number of cells showed no decline at all. However, the number of cells improved compared to the control group (Figure S6). This outcome illustrates that CNC-AN freeze-dried powder did not influence the viability of HaCaT cells, which shows assured biosecurity. Using ROS level, comet assay, and cell immunofluorescence in *vitro*, we also demonstrated the protection of UVB-induced damage in lip gloss and lipstick. In particular, one kind of common sunscreen (product name: Neutrogena SPF 30 Dry-touch Sunscreen) was used as a positive control (≈15 *μ*m thickness). The level of ROS was detected as green fluorescence proportional intensity. After UVB irritation, the intracellular ROS level in HaCaT cells was significantly different compared with control, sunscreen, lip gloss, and lipstick groups. The ROS level in the lip gloss and lipstick groups showed no significant differences compared to the control and sunscreen groups, suggesting that CNC-AN, lip gloss, and lipstick can protect cells against UVB-induced oxidative stress (Figure S7). Then, we measured the single and double-strand break of DNA in cells using the comet assay [22]. The percentage of DNA content in the comet tail might represent the levels of DNA damage. Specifically, DNA% tail was higher in the UVB group, while lip gloss and lipstick groups exhibited no change compared with the control and sunscreen groups (Figures 4(a) and 4(b)). *γ*H2AX is a marker of nucleus damage [44]. Subsequently, we applied cell immunofluorescence to detect the expression of *γ*H2AX in order to demonstrate the protection of CNC-AN gel and lipstick from UVB-induced nucleus impairment. It is apparent that while the expression of *γ*H2AX improved a lot in the UVB group, the lip gloss and lipstick groups showed no differences compared with the control and sunscreen groups (Figures 4(c)–4(f)). From comet assay and cell immunofluorescence H2AX, the protection of nucleus damage was evident.

### 3.7. Evaluation of Anti-UVB Damage Activity *In Vivo*

**Figure 5:**
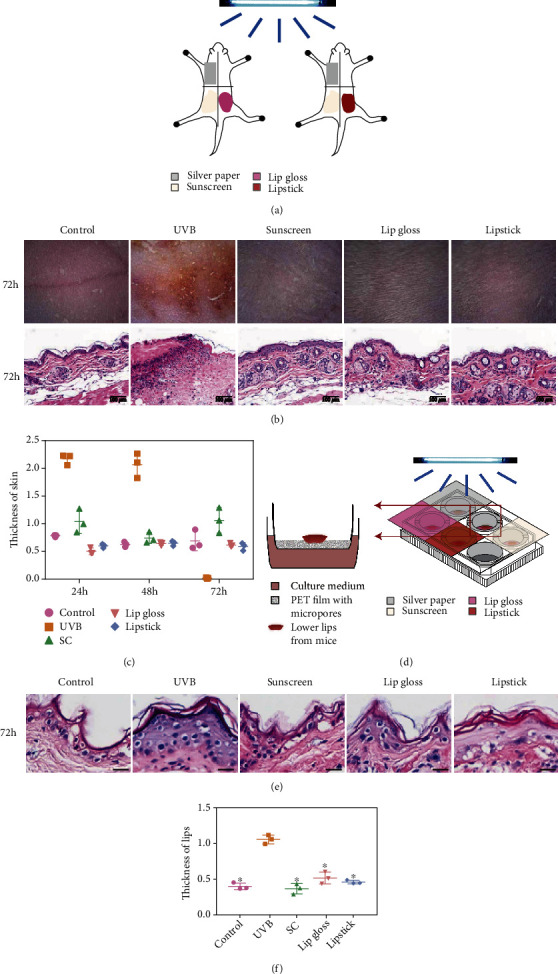
Dermatoscope and histology of dorsal mouse skin applying different topical interventions after UV irradiation. (a) Model diagram of mouse protection against UVB irradiation. (b) The dermatoscope and hematoxylin/eosin staining of dorsal skin in mice. (c) Thickness of skin in mice. ^∗^*P* < 0.05, the difference has statistical significance. Scale bars, 100 *μ*m. (d) Model diagram of organotypic culture showing lip protection against UVB irradiation. (e) Hematoxylin/eosin staining of the lip in each group. ^∗^*P* < 0.05, the difference has statistical significance. Scale bars, 25 *μ*m.

We further performed protection in *vivo* research to observe the efficiency of CNC-AN, lip gloss, and lipstick in Balb/c mice. We divided the back of these mice into 4 sections, including control, UVB, sunscreen, and experimental groups (Figure 5(a)). One of them was dealt with gel, while another was dealt with lipstick in the experimental group (every section was covered with 1 cm × 1 cm × 100 *μ*m sunscreen, lip gloss, and lipstick, except the control and UVB groups. In addition, the control group was covered with a silver paper). These mice were exposed to UVB radiation with 5040 mJ/cm^2^ for single use. After radiation, the dermatoscope and hematoxylin/eosin staining of dorsal skin in each group was executed at 24, 48, and 72 h, respectively (Figures 5(b) and 5(c), S8, S9). In the images of the dermatoscope, UVB-induced damage in the skin after 24 and 48 h was illegible (Figure S8). However, after 72 h, the UVB group manifested clearly erythema, blister, erosion, and crust. Notably, the erythema value in the UVB group at 72 h was much higher than in other groups (Figure 5(b)). The CNC-AN gel and lipstick group conformed to the control and sunscreen groups, which identified the efficient protection against UVB. Based on the hematoxylin/eosin staining of the dorsal skin, the thickness of the skin was increased clearly at 24 and 48 h in the UVB group compared with other groups (Figure S9). In addition, the dermis was fully inflammatory infiltrate while sebaceous structures looked hypertrophic, especially at 72 h in the UVB group. Furthermore, the skin disappeared completely at 72 h after UVB radiation, with alteration of the skin replaced by blisters and crust (Figures 5(b) and 5(c)). Similarly, the protection of the CNC-AN, lip gloss, and lipstick groups showed no substantial change relative to the control and sunscreen groups.

Moreover, the epidermal and sebaceous hypertrophy effect in the skin protected by CNC-AN, lip gloss, and lipstick was significantly inhibited even compared with the sunscreen group, which appeared slightly hypertrophic relative to the control group. In this study, we applied lip culture in BALB/C mice so as to confirm the protection of CNC-AN, lip gloss, and lipstick for lips. To begin with, lip explants were incubated epidermal-side-up on a transwell in 6-well plates. Briefly, 5 wells were assigned to control, UVB, sunscreen, lip gloss, and lipstick groups, respectively (Figure 5(d)). Every well was covered with 100 *μ*m sunscreen, lip gloss, and lipstick, except for the control and UVB groups. The control group was covered with a silver paper. Then, a 6-well plate was exposed to UVB radiation with 5040 mJ/cm^2^ for single use. After radiation, owing to evidence from dorsal skin, hematoxylin/eosin staining of the lip in each group was performed at 72 h (Figure 5(e)). The thickness of the lip increased apparently at 72 h in the UVB group relative to other groups. Interestingly, the protection of the CNC-AN gel and lipstick group showed significant protection compared with the control and sunscreen groups. Both organotypic cultures and in *vivo* experiments manifested excellent anti-UVB protection of CNC-AN gel and lipstick.

### 3.8. Human Evaluation

**Figure 6:**
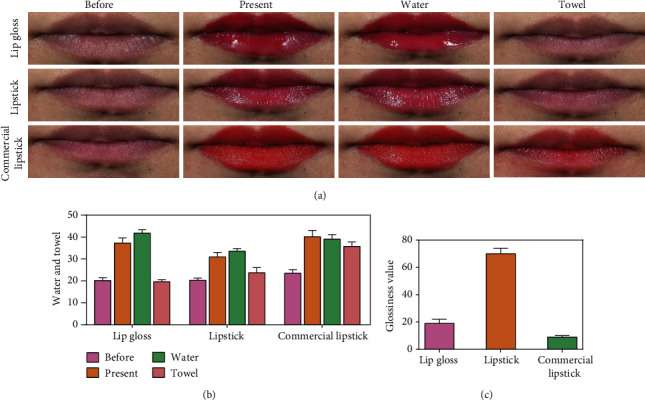
(a) VISIA images of lip gloss, lipstick, and commercial lipstick cleansing after washing with water or wiping with a wet towel. (b) The values of VISIA images. (c) The glossiness value before and after applying lipsticks.

The bioadhesion and retention of CNC-AN lip gloss and lipstick in human skin were examined using CIE 3D space system. After application, the changes in *L*∗ and *a*∗ values for 8 h were measured at 9 time points. There were no significant differences in the values of *L*∗ and *a*∗ among lip gloss, lipstick, and commercial lipsticks, showing excellent bioadhesion and retention in agreement with commercial lipstick (Figure S11). To explore the water persistence and easy wiping with a wet towel of CNC-AN gel and lipstick in the human lip, VISIA was applied to collect photographs at different time points (Figure 6(a)). We recorded the glossiness value before and after applying lip gloss, lipstick, and commercial lipstick. Our findings revealed that CNC-AN gel and lipstick were better than commercial lipstick (Figure 6(c)). After water washing, all of these three gel and lipsticks were not removed, thus indicating excellent water persistence. Further confirmation, after cleaning with a wet towel, lip gloss and lipstick groups were completely discarded, while the commercial lipstick group left a large amount of residual lipstick on the lips (Figure 6(b)). The characteristics of water persistence and easy wiping with a wet towel were also proved by the use of a dermatoscope (Figure S10). In conclusion, the characteristics of wonderful bioadhesion and water persistence are qualified for a product as lipstick. Furthermore, after applying the CNC, the gel and lipstick convert to easy wiping with a wet towel, offering those people with skin diseases, such as rosacea, contact dermatitis, acne, and some other sensitive skin that are too fragile to use a facial cleanser. Finally, it is convenient to clean it at any time such as before meals, for the reason that lipstick eating with food is harmful to health [45].

## 4. Conclusion

In summary, the protection of UV-induced lip damage has recently attracted greater research attention. Inspired by the traditional Chinese method, we applied anthocyanins to formulate lipsticks. Combining our previous research, we grafted CNC with AN to form a biomolecule characterized by enhanced bioadhesion, retention, and easy wiping characteristics. Surprisingly, CNC improved the SPF value of our new lipstick beyond our expectation. Therefore, our new creativeness provides a series of natural-sourced, environmentally friendly raw materials for more widespread use in the cosmetic industry. Finally, our lipstick has potential for commercial application owing to its natural-sourced, environmentally friendly, excellent bioadhesion, water resistance, easy wiping with a wet towel, and excellent UV-shielding features that enable this sunscreen lipstick to fill a gap in the market.

## Data Availability

The data used to support the findings of this study are included within the article and supplementary information files.

## Additional Points

*Rationale.* Cellulose nanocrystals reinforced enhancement of UV absorption and shielding properties of anthocyanin by improving specific extinction value. *Objective*. Safer bio-based sunscreens of cellulose enveloped with anthocyanin were bionic designed evaluated *in vitro* and *in vivo*.

## Supplementary Materials

Supplementary Materials The supplementary description includes graph abstract of our research, the method for preparation and characterization of anthocyanin, binding capacity experiments, scanning electron microscopic (SEM) images of CNCs and CNCs grafting AN, continuous shooting of CNCs, AN and CNCs-AN, UV absorption, cell viability assay, ROS fluorescence level, dermatoscope and H&E staining of mouse dorsal skin, dermatoscope, and CIE 3D in forearm skin in human.
