# Supplementary material for: Self-Assembly of Cellulose Nanocrystals and Organic Colored Pigments as Reinforcement Matrix of Lipstick for Enhancing SPF
Source: Oxid Med Cell Longev. 2022 Feb 9;2022:2422618. doi: 10.1155/2022/2422618 (PMC8850073; doi:10.1155/2022/2422618)
Supplement: Supplementary Materials — The supplementary description includes graph abstract of our research, the method for preparation and characterization of anthocyanin, binding capacity experiments, scanning electron microscopic (SEM) images of CNCs and CNCs grafting AN, continuous shooting of CNCs, AN and CNCs-AN, UV absorption, cell viability assay, ROS fluorescence level, dermatoscope and H&E staining of mouse dorsal skin, dermatoscope, and CIE 3D in forearm skin in human. [file 2422618.f1.zip › Figure Si.docx]

**Supporting Information**

*
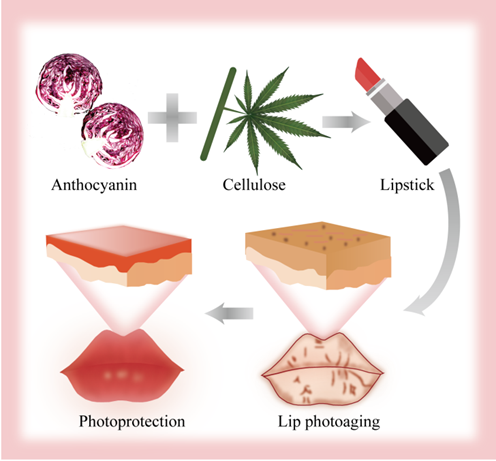
*

Graph Abstract

*Preparation and Characterization of Anthocyanin:*

AN was extracted from a variety of plants or buying from the manufacturer.AN were extracted by ethanol from blueberries, plums, red cabbage, dragon fruit, or purple onion﻿ purchased from the local market. Wash fresh red cabbage (Cyanidin-3-O-glucoside chloride as standard, HPLC≥98%), crush 50 s, add deionized water 1:5 (W/V) The following conditions: pectinase 0.0013 ml/100g, 55.6℃, pH 4.1, and 50 min. Then, the temperature was rapidly increased to 95℃ for 5 min to passivate pectinase and quickly cooled to room temperature. Filtered it to obtain a fuchsia clear pigment solution. Finally freeze dry it to obtain freeze-dried jelly. HPLC analysis was applied by using waters e2695 (Waters, USA) instrument and PDA detector (2998, Waters, USA) to detect the content of AN. To detect the content of self-extracting AN in red cabbage, high performance liquid chromatography (HPLC) analysis shows the average concentration of AN is 160.63±0.05 μg/ml (Figure S1).The treatments of blueberries, plums, dragon fruit, and purple onion were in the same way. The same applied commercialized AN from Lgberry Co,Ltd with an 5 wt.% AN content.


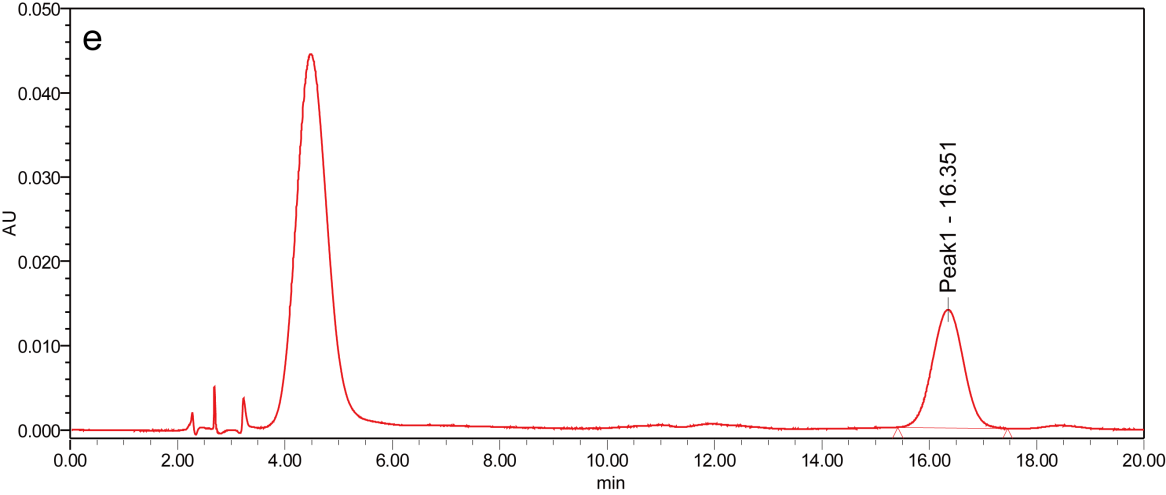


Figure S1. High performance liquid chromatography (HPLC) analysis was applied to detect the content of AN purple cabbage.

﻿

*Binding capacity experiments:*

Standard curve prepared with AN (Cyanidin-3-O-glucoside chloride, HPLC≥98%) at 530 nm. Approximately 500 mg CNC were incubated in 1–2 mg/ml AN solutions for 2 h. Tubes were constantly mixed at 100 rpm on an orbital shaker, in a dark room at 4 °C. Control samples without CNC were also incubated under the same conditions. After given contact times, the absorbance was measured under study using a UV–Vis spectrophotometer. The amount of bound AN was quantified by subtracting the amount of AN in the control solutions from that in the treated sample solutions. Although the amounts of bound AN increased with a low-dose regimen, this does not mean that all of the available AN in the solution will interact with CNC after a high concentration. The highest concentration reached up to 60% w/w of AN to CNC (Figure S2).


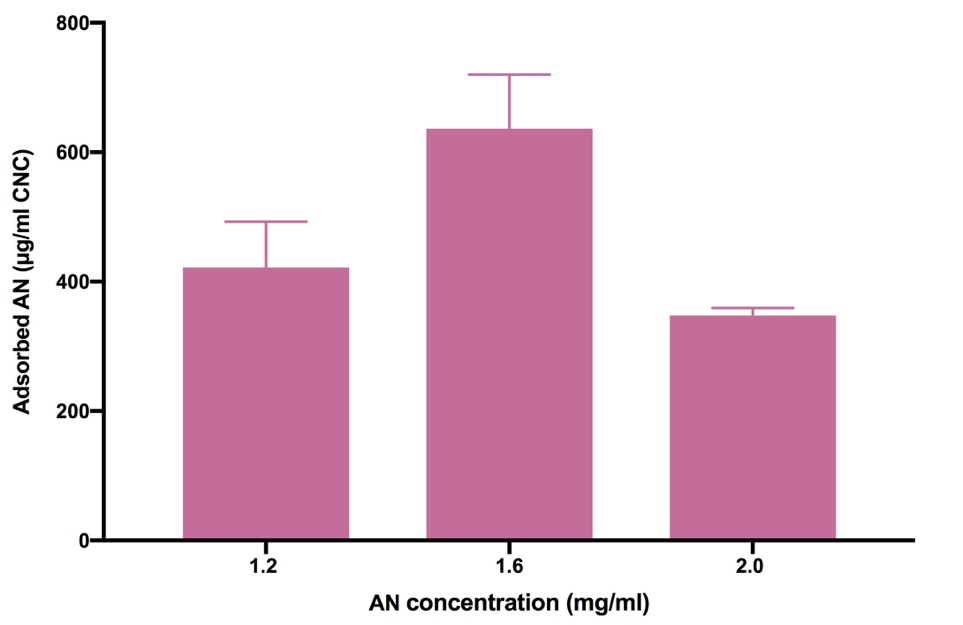


Figure S2. The binding capacity of AN to CNC.


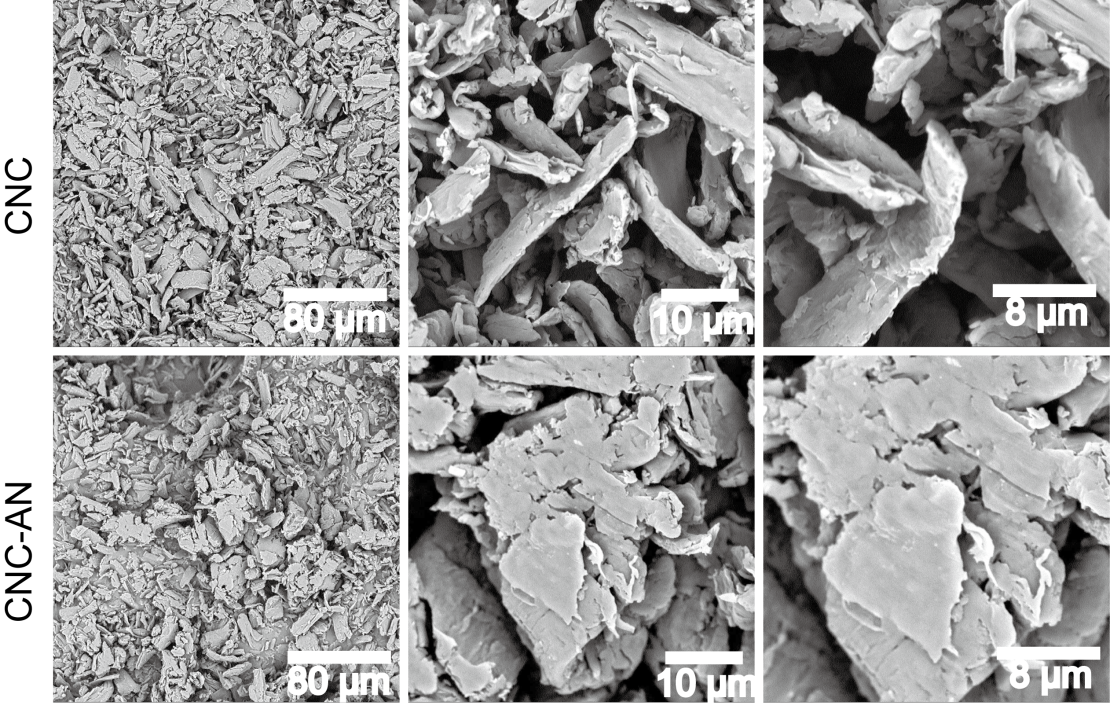


Figure S3. Scanning electron microscopic (SEM) images of CNCs and CNCs grafting AN.


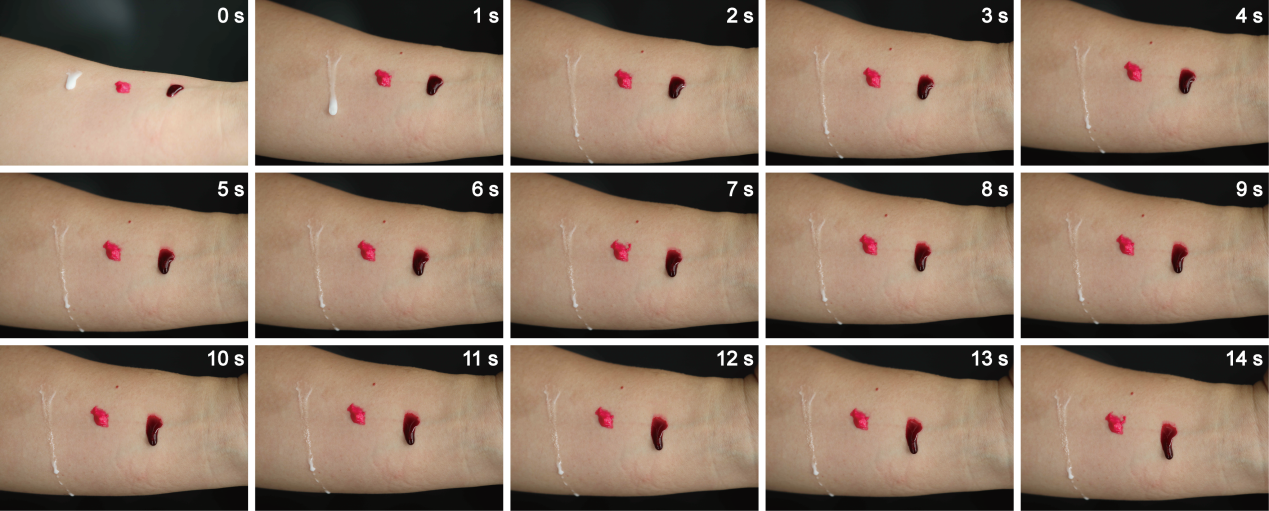


Figure S4. Bioadhesive characteristic of CNCs, AN and CNCs grafting AN applying continuous shooting for 15 s.


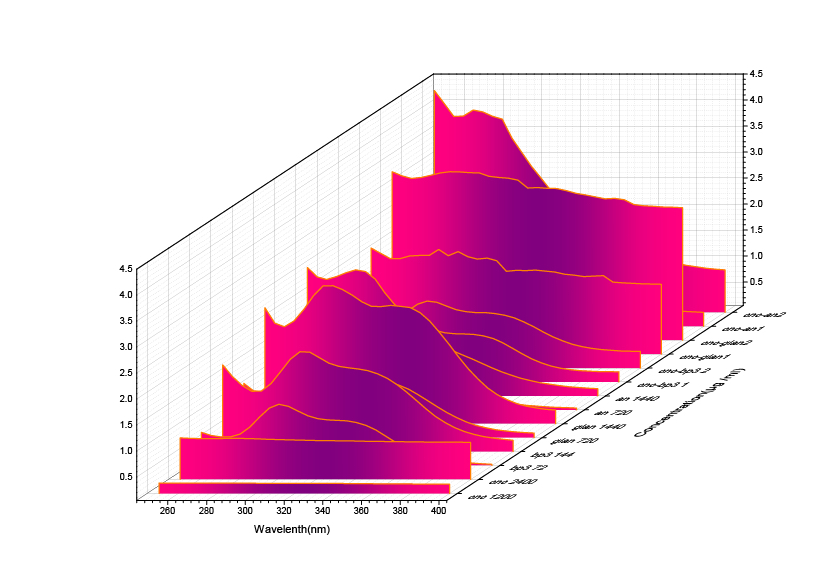


Figure S5. The UV absorption of 1200 μg/ml CNC, 2400 μg/ml CNC, 720 μg/ml AN(commercialized AN), 1440 μg/ml AN, 720 μg/ml GLAN(extracting AN from red cabbage), 1440 μg/ml GLAN, 72 μg/ml BP-3 (benzophenone-3), 144 μg/ml BP-3, CNC-AN1(1200 μg/ml CNC binding to 720 μg/ml AN), CNC-AN2(2400 μg/ml CNC binding to 1440 μg/ml AN), CNC-GLAN1(1200 μg/ml CNC binding to 720 μg/ml GLAN), CNC-GLAN2(2400 μg/ml CNC binding to 1440 μg/ml GLAN),CNC-BP3 1(1200 μg/ml CNC binding to 72 μg/ml BP3), and CNC-BP3 2(2400 μg/ml CNC binding to 144 μg/ml BP3)


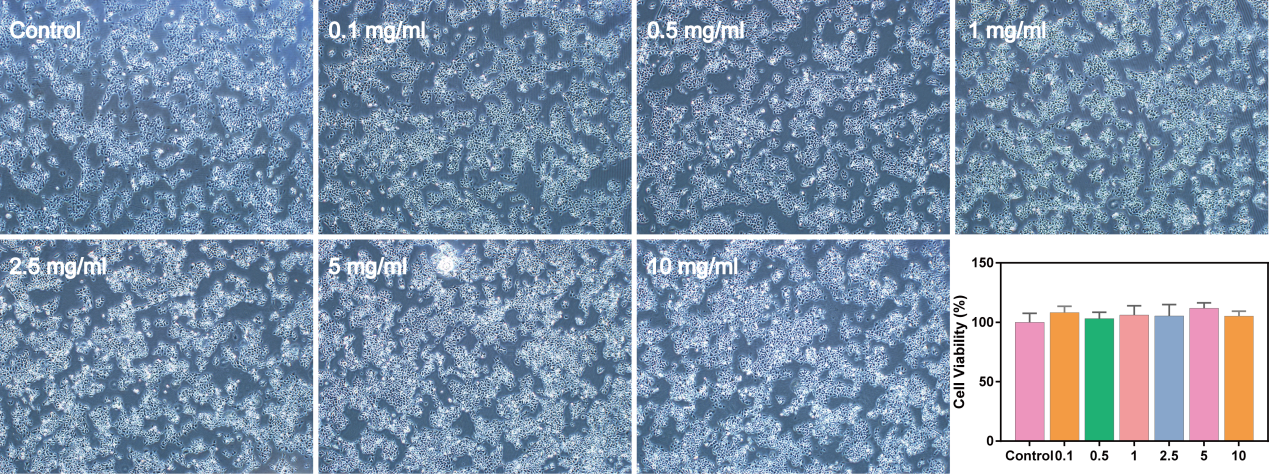


Figure S6. Cell viability assay of CNC-AN freeze-dried powder.


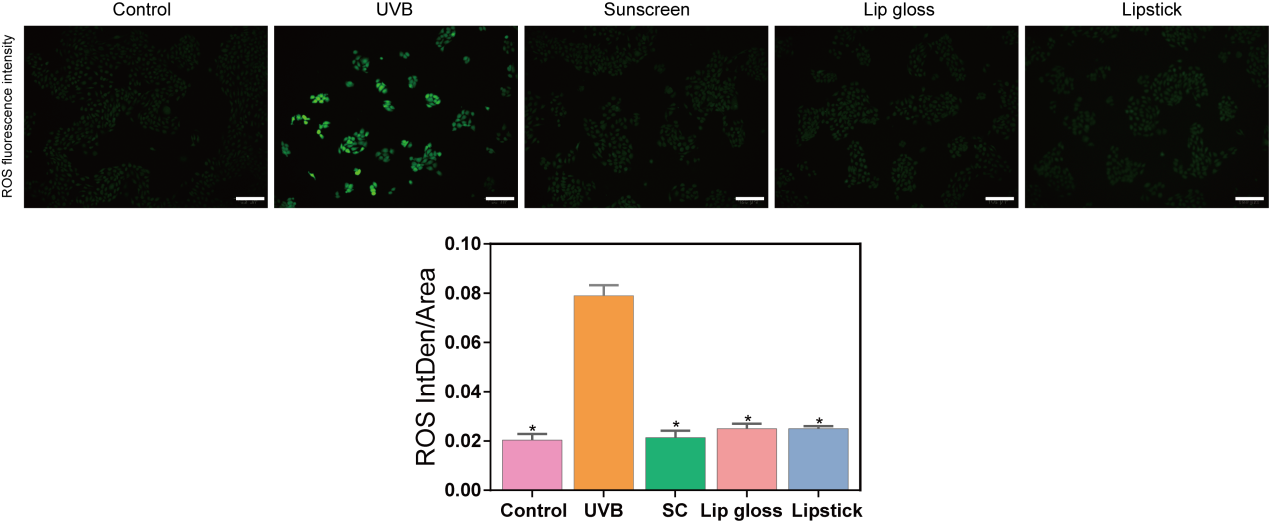


Figure S7. ROS fluorescence level in Control, UVB, sunscreen, CNCs+AN lip gloss and CNC-AN lipstick group and statistical figure of ROS fluorescence level. *, P＜0.05, the difference has statistically significance. Scale bars, 50 μm.


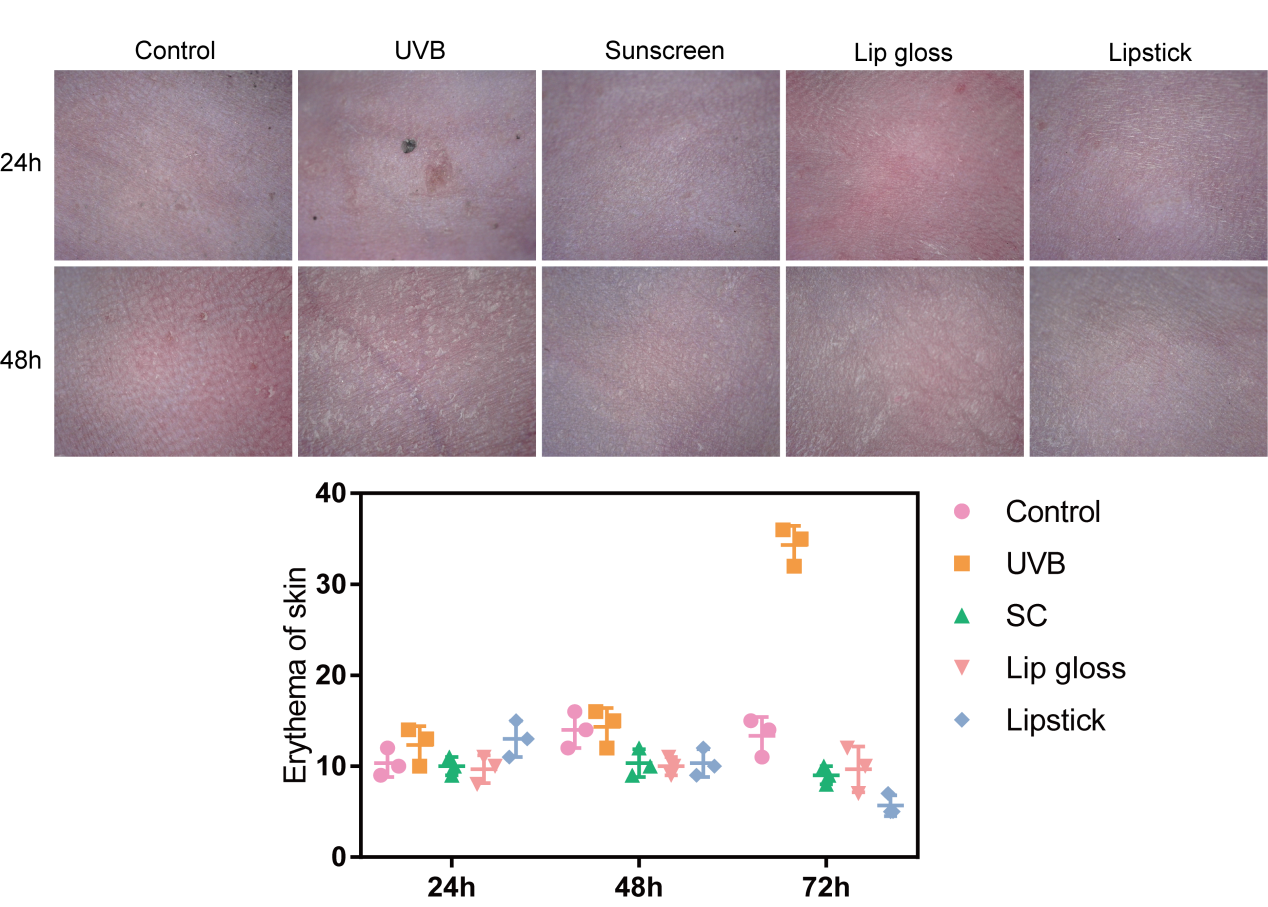


Figure S8. Dermatoscope of dorsal mouse skin applying different topical interventions after UV irradiation at 24 h and 48 h. Relative erythema value of dorsal skin in mice. *, P＜0.05, the difference has statistically significance.


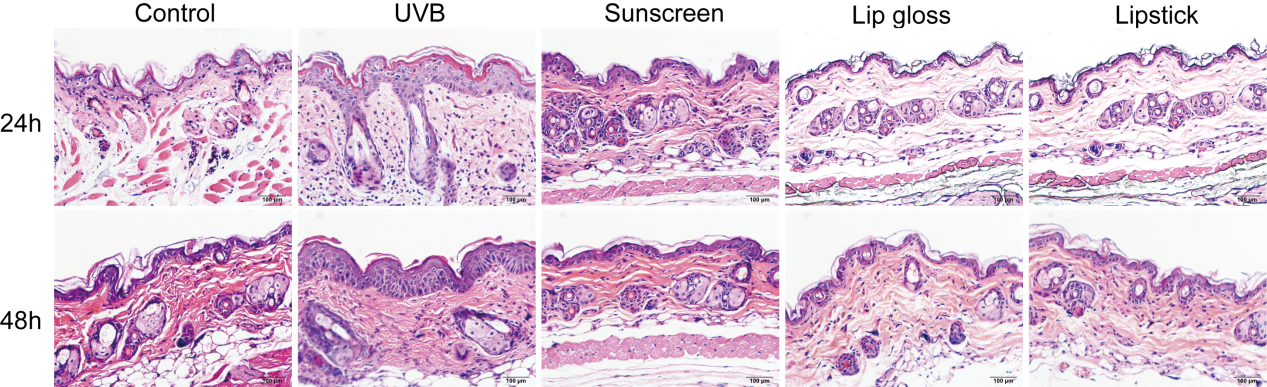


Figure S9. The dermatoscope and hematoxylin/eosin staining of dorsal skin in mice. Scale bars, 100 μm.


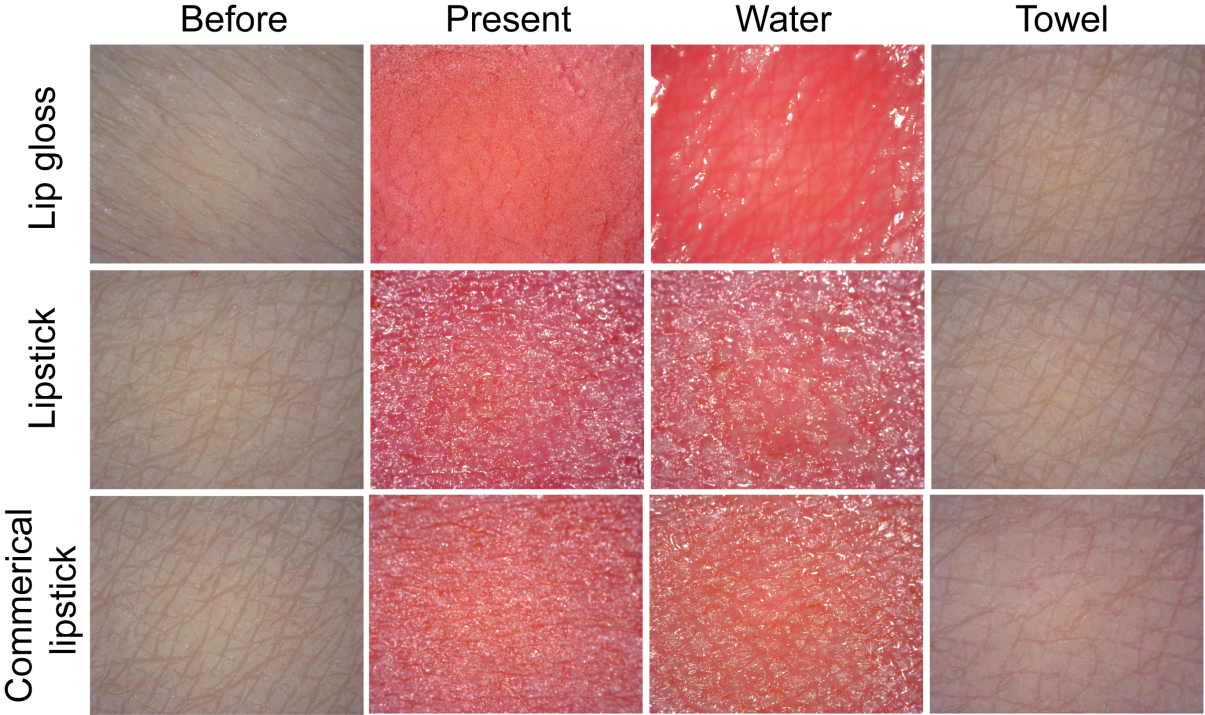


Figure S10. The dermatoscope of CNCs-g-PLA lip gloss, lipstick and commercial lipstick cleanising after washing with water or wiping with a wet towel in forearm skin in human.


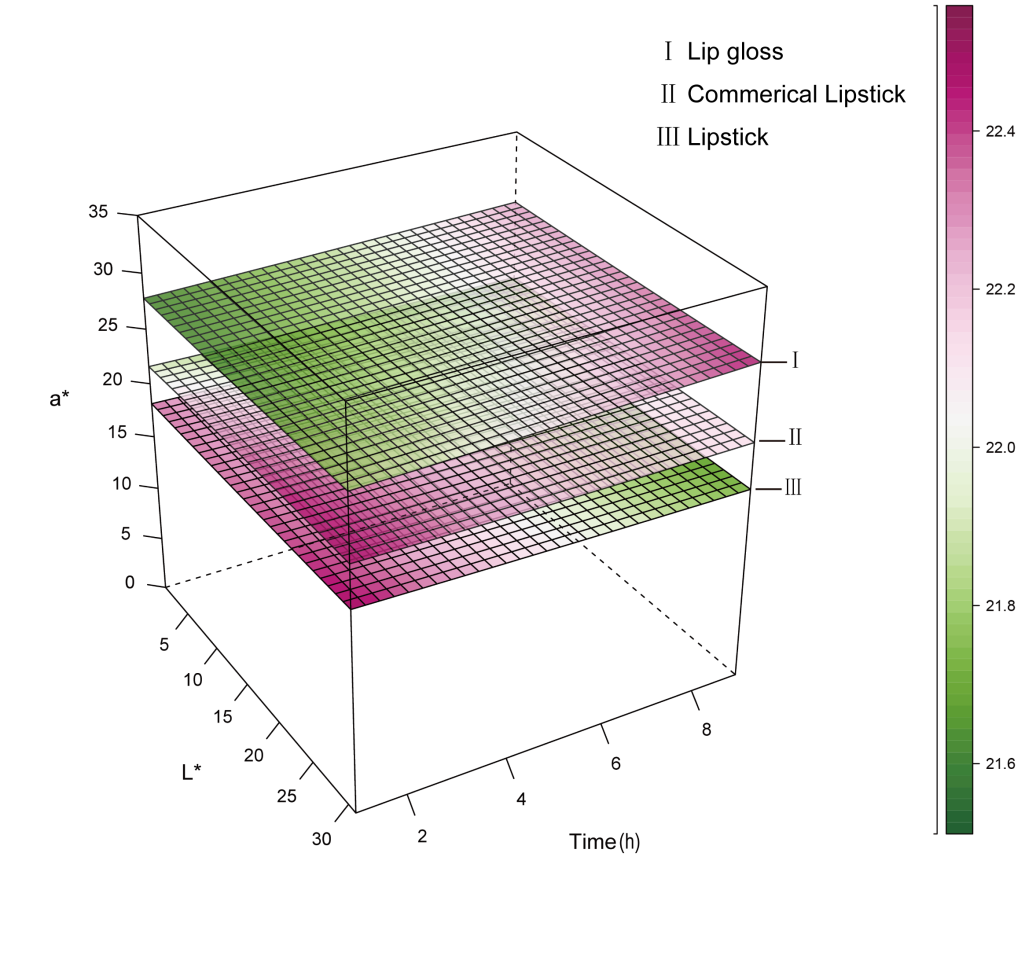


Figure S11. The CIE 3D space system was used to evaluate the bioadhesion and retention of lipstick and lip gloss in human.
