# Supplementary material for: Self-Assembly of Cellulose Nanocrystals and Organic Colored Pigments as Reinforcement Matrix of Lipstick for Enhancing SPF
Source: Oxid Med Cell Longev. 2022 Feb 9;2022:2422618. doi: 10.1155/2022/2422618 (PMC8850073; doi:10.1155/2022/2422618)
Supplement: Supplementary Materials — The supplementary description includes graph abstract of our research, the method for preparation and characterization of anthocyanin, binding capacity experiments, scanning electron microscopic (SEM) images of CNCs and CNCs grafting AN, continuous shooting of CNCs, AN and CNCs-AN, UV absorption, cell viability assay, ROS fluorescence level, dermatoscope and H&E staining of mouse dorsal skin, dermatoscope, and CIE 3D in forearm skin in human. [file 2422618.f1.zip › Table Si.docx]

Table S1 Absorption peaks (λmax) and molar extinction coefficients (ε) of BP-3 and AN

| ﻿Substance | ﻿AN | BP-3 |
| --- | --- | --- |
| ﻿UV absorption λmax (nm) | 280 | 290 |
| ﻿ε (cm^−1^ M^−1^) | 761.07±38.26 | 4229.01±651.98 |

Table S2 ﻿ Formulation of lipstick

| ﻿Ingredient | ﻿ | Contents |
| --- | --- | --- |
| ﻿Soft oil | Olive Oil  Vaseline  Glyceryl stearate | 4.8g  0.3g  0.6g |
| ﻿ | Tridecanol triphenyl ester  Bis-diglyceryl polyacyladipate-2 | 0.3g  0.6g |
| Hard oil | Ozocerite  Microcrystalline wax  Bee wax | 0.9g  0.9g  0.3g |
| CNC-AN |  | 1g |

Table S3 ﻿ ﻿SPF obtained from BASF simulator and from SPF 290S

| ﻿ Formulation | ﻿﻿SPF from BASF simulator | SPF 290S |
| --- | --- | --- |
| cHEC | NP | 0.93±0.03 |
| ﻿5% HMS-cHEC | 3.2 | 3.41±0.23 |
| ﻿5% OCR-cHEC | 6.0 | 6.23±0.12 |
| 4% MBC-cHEC | 6.6 | 6.52±0.32 |
| 5% BP-3-cHEC | 7.4 | 7.32±0.53 |
| CNC-cHEC | NP | 0.37±0.01 |
| 5% CNC-BP-3-cHEC | NP | 7.60±0.98 |
| 5% AN-cHEC | NP | 2.16±0.62 |
| 5% CNC-AN-cHEC  (lip gloss) | NP | 22.12±1.58 |
| lipstick without CNC-AN | NP | 6.19±1.76 |
| lipstick with CNC-AN | NP | 30.33±2.77 |
| Commerical lipstick | NP | 12.22±0.23 |

﻿NP: not performed; SPF: sun protection factor
